# *fac*-Tricarbon­yl(pyridine-κ*N*)(1,1,1-trifluoro­acetyl­acetonato-κ^2^ *O*,*O*′)rhenium(I)

**DOI:** 10.1107/S160053681104476X

**Published:** 2011-10-29

**Authors:** Hendrik G. Visser, Andreas Roodt, Amanda-lee Volmink, Gerdus Kemp

**Affiliations:** a Department of Chemistry, University of the Free State, PO Box 339, Bloemfontein 9300, South Africa; b Petlabs, Little Company of Mary Hospital, Pretoria 0001 9300, South Africa

## Abstract

In the title compound, [Re(C_5_H_4_F_3_O_2_)(C_5_H_5_N)(CO)_3_], the Re^I^ atom is six-coordinated owing to bonding by three carbonyl ligands arranged in a *fac* configuration, two O atoms from the bidentate 1,1,1-trifluoro­acetyl­acetonate ligand and an N atom from a pyridine ligand. In the crystal, the mol­ecules pack in layers, diagonally, in a head-to-tail fashion across the *ab* plane. These layers are stabilsed by inter­molecular C—H⋯O and C—H⋯F hydrogen bonds.

## Related literature

For the synthesis of the Re(I)-tricarbonyl synthon, see: Alberto *et al.* (1996 ▶). For related rhenium–tricarbonyl complexes, see: Brink *et al.* (2009 ▶, 2011 ▶); Mundwiler *et al.* (2004 ▶); Schutte *et al.* (2010 ▶). For a review on structure–reactivity relationships, see: Roodt *et al.* (2011 ▶).
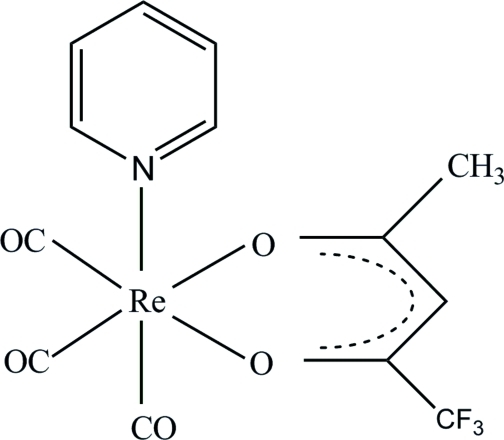


## Experimental

### 

#### Crystal data

[Re(C_5_H_4_F_3_O_2_)(C_5_H_5_N)(CO)_3_]
*M* *_r_* = 502.41
Monoclinic, 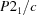

*a* = 15.561 (2) Å
*b* = 6.982 (3) Å
*c* = 14.082 (5) Å
β = 103.271 (5)°
*V* = 1489.1 (9) Å^3^
*Z* = 4
Mo *K*α radiation
μ = 8.22 mm^−1^
*T* = 100 K
0.15 × 0.10 × 0.03 mm

#### Data collection

Bruker X8 APEXII 4K Kappa CCD diffractometer
Absorption correction: multi-scan (*SADABS*; Bruker, 2004 ▶) *T*_min_ = 0.328, *T*_max_ = 0.778
17351 measured reflections
3603 independent reflections
3104 reflections with *I* > 2σ(*I*)
*R*_int_ = 0.040

#### Refinement

*R*[*F* ^2^ > 2σ(*F* ^2^)] = 0.025
*wR*(*F* ^2^) = 0.059
*S* = 1.06
3603 reflections
209 parameters
H-atom parameters constrained
Δρ_max_ = 1.43 e Å^−3^
Δρ_min_ = −1.09 e Å^−3^

### 

Data collection: *APEX2* (Bruker, 2010 ▶); cell refinement: *SAINT-Plus* (Bruker, 2004 ▶); data reduction: *SAINT-Plus*; program(s) used to solve structure: *SHELXS97* (Sheldrick, 2008 ▶); program(s) used to refine structure: *SHELXL97* (Sheldrick, 2008 ▶); molecular graphics: *DIAMOND* (Brandenburg & Putz, 2005 ▶); software used to prepare material for publication: *WinGX* (Farrugia, 1999 ▶).

## Supplementary Material

Crystal structure: contains datablock(s) global, I. DOI: 10.1107/S160053681104476X/ng5257sup1.cif

Structure factors: contains datablock(s) I. DOI: 10.1107/S160053681104476X/ng5257Isup2.hkl

Additional supplementary materials:  crystallographic information; 3D view; checkCIF report

## Figures and Tables

**Table 1:** Hydrogen-bond geometry (Å, °)

*D*—H⋯*A*	*D*—H	H⋯*A*	*D*⋯*A*	*D*—H⋯*A*
C2—H2⋯F3^i^	0.93	2.55	3.407 (6)	153
C22—H22⋯O1^ii^	0.93	2.58	3.360 (5)	142

Symmetry codes: (i) 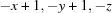; (ii) 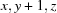.

**Fig. 1.**
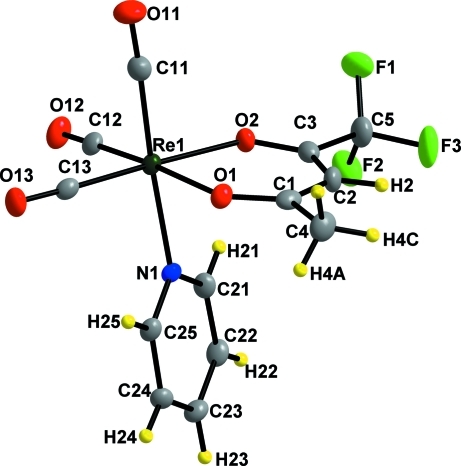
Molecular structure of the title compound. Displacement ellipsoids are drawn at the 50% probability level.

**Fig. 2.**
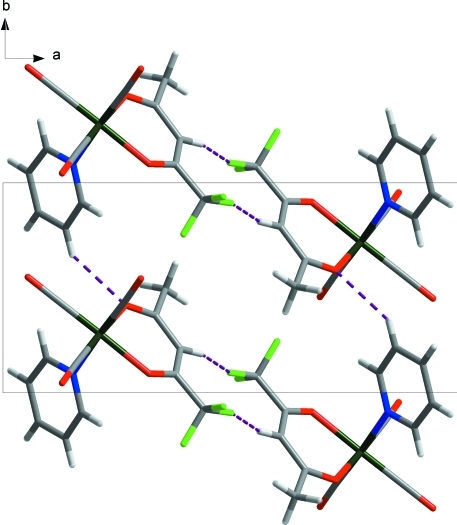
Packing and hydrogen bonding as observed across the ab plane. Symmetry codes: (i) 1 - x, 1 - y, -z, (ii) x, y + 1, z.

## supplementary crystallographic information

### Comment

This work forms part of our ongoing research in structure/reactivity relationships (Roodt *et al.*, 2011) and the applications of rhenium- tricarbonyl complexes in the radiopharmaceutical industry (Brink *et al.*, 2009, 2011; Schutte *et al.*, 2010).

In the title Rhenium(I) compound, [Re(C_5_F_3_H_4_O_2_)(CO)_3_(py)], each rhenium atom is six-coordinated to three carbonyl ligands, two oxygen atoms from the bidentate 1,1,1-trifluoroacetylacetonato ligand and a nitrogen atom from a pyridine ligand to form a slightly distorted octahedron (see Figure 1). This is illustrated by the small deviations from 90 °, with the O1—Re1—N1 being the furthest outlier (82.72 (11) Å. All the bonding distances and angles are considered normal (Mundwiler *et al.* 2004); Brink *et al.* 2009, 2011). The three carbonyl ligands are arranged in a facial configuration around the Re atom.

Interestingly, it does not seem as if the electronwithdrawing properties of the fluorine molecules on the bidentate ligand backbone have any effect on bonding distances in the molecule (as opposed to the methyl group). The *trans* Re—C bonding distances are exactly the same (Re1—C12; Re1—C13; 1.906 (4) Å) while the Re1—O2 distance of 2.117 (3) Å is similar to Re1—O1 within experimental error (2.135 (3) Å).

The molecules pack in layers, diagonally, in a head-to-tail fashion across the *ab* plane. These layers are stabilsed by intermolecular CH–O and CH–F hydrogen bonds (see Figure 2).

### Experimental

[Re(CO)_3_(Br)_3_] (500 mg; 0.648 mmol) was prepared according to the method of Alberto (Alberto *et al.*, 1996) and was dissolved in 10 ml water (pH 2.2) while stirring for 30 min. To this solution, AgNO_3_ (330 mg; 1.945 mmol) was added and stirred for 24 h at room temperature. The precipitate, AgBr, was filtered off after which trifluoroacetylacetone (0.1 g; 0.649 mmol) was added to the filtrate and stirred for another 48 hrs. To the yellow solution, pyridine (0.0512 g; 0.648 mmol) was added and stirred for 10 min. at room temperature. A bright yellow precipitate formed which was filtered off and recrystallized from acetone (3 ml). Yellow needles were obtained (yield = 0.292 g; 89%)

### Refinement

The methine and methylene H atoms were placed in geometrically idealized positions at C—H = 0.93 and 0.97 Å, respectively and constrained to ride on their parent atoms, with *U*_iso_(H) = 1.2*U*_eq_(C). The highest peak is located 0.79 Å from Re1 and the deepest hole is situated 0.95 Å from Re1.

### Crystal data

**Table:**

[Re(C_5_H_4_F_3_O_2_)(C_5_H_5_N)(CO)_3_]	*F*(000) = 944
*M**_r_* = 502.41	*D*_x_ = 2.241 Mg m^−^^3^
Monoclinic, *P*2_1_/*c*	Mo *K*α radiation, λ = 0.71073 Å
Hall symbol: -P 2ybc	Cell parameters from 7262 reflections
*a* = 15.561 (2) Å	θ = 2.7–28.2°
*b* = 6.982 (3) Å	µ = 8.22 mm^−^^1^
*c* = 14.082 (5) Å	*T* = 100 K
β = 103.271 (5)°	Needle, yellow
*V* = 1489.1 (9) Å^3^	0.15 × 0.1 × 0.03 mm
*Z* = 4	

### Data collection

**Table:**

Bruker X8 APEXII 4K Kappa CCD diffractometer	3603 independent reflections
Radiation source: fine-focus sealed tube	3104 reflections with *I* > 2σ(*I*)
graphite	*R*_int_ = 0.040
φ and ω scans	θ_max_ = 28°, θ_min_ = 2.7°
Absorption correction: multi-scan (*SADABS*; Bruker, 2004)	*h* = −20→20
*T*_min_ = 0.328, *T*_max_ = 0.778	*k* = −9→9
17351 measured reflections	*l* = −18→16

### Refinement

**Table:**

Refinement on *F*^2^	Primary atom site location: structure-invariant direct methods
Least-squares matrix: full	Secondary atom site location: difference Fourier map
*R*[*F*^2^ > 2σ(*F*^2^)] = 0.025	Hydrogen site location: inferred from neighbouring sites
*wR*(*F*^2^) = 0.059	H-atom parameters constrained
*S* = 1.06	*w* = 1/[σ^2^(*F*_o_^2^) + (0.0265*P*)^2^ + 2.5868*P*] where *P* = (*F*_o_^2^ + 2*F*_c_^2^)/3
3603 reflections	(Δ/σ)_max_ = 0.001
209 parameters	Δρ_max_ = 1.43 e Å^−^^3^
0 restraints	Δρ_min_ = −1.09 e Å^−^^3^

### Special details

**Table:**

Experimental. The intensity data were collected on a Bruker X8 ApexII 4 K Kappa CCD diffractometer using an exposure time of 40 s/frame. A total of 1709 frames were collected with a frame width of 0.5° covering up to θ = 28.39° with 99.9% completeness accomplished.
Geometry. All s.u.'s (except the s.u. in the dihedral angle between two l.s. planes) are estimated using the full covariance matrix. The cell s.u.'s are taken into account individually in the estimation of s.u.'s in distances, angles and torsion angles; correlations between s.u.'s in cell parameters are only used when they are defined by crystal symmetry. An approximate (isotropic) treatment of cell s.u.'s is used for estimating s.u.'s involving l.s. planes.
Refinement. Refinement of *F*^2^ against ALL reflections. The weighted *R*-factor *wR* and goodness of fit *S* are based on *F*^2^, conventional *R*-factors *R* are based on *F*, with *F* set to zero for negative *F*^2^. The threshold expression of *F*^2^ > 2σ(*F*^2^) is used only for calculating *R*-factors(gt) *etc*. and is not relevant to the choice of reflections for refinement. *R*-factors based on *F*^2^ are statistically about twice as large as those based on *F*, and *R*- factors based on ALL data will be even larger.

### Fractional atomic coordinates and isotropic or equivalent isotropic displacement parameters (Å^2^)

**Table:**

	*x*	*y*	*z*	*U*_iso_*/*U*_eq_	
C1	0.3352 (3)	0.1319 (6)	0.0313 (3)	0.0209 (9)	
C2	0.3897 (3)	0.2909 (6)	0.0641 (3)	0.0233 (9)	
H2	0.436	0.3153	0.0341	0.028*	
C3	0.3793 (3)	0.4120 (6)	0.1371 (3)	0.0211 (9)	
C4	0.3568 (3)	0.0060 (7)	−0.0476 (3)	0.0289 (10)	
H4A	0.3044	−0.0147	−0.0978	0.043*	
H4B	0.3791	−0.1148	−0.0199	0.043*	
H4C	0.4006	0.0677	−0.0751	0.043*	
C5	0.4426 (3)	0.5780 (7)	0.1659 (4)	0.0292 (10)	
C11	0.2715 (3)	0.0553 (6)	0.2774 (3)	0.0224 (9)	
C12	0.1608 (3)	0.3619 (6)	0.2583 (3)	0.0193 (8)	
C13	0.1142 (3)	0.0508 (6)	0.1529 (3)	0.0178 (8)	
C21	0.1734 (3)	0.5963 (6)	0.0479 (3)	0.0189 (8)	
H21	0.2129	0.6478	0.1015	0.023*	
C22	0.1372 (3)	0.7145 (5)	−0.0285 (3)	0.0205 (9)	
H22	0.1524	0.8436	−0.0264	0.025*	
C23	0.0777 (3)	0.6403 (6)	−0.1093 (3)	0.0205 (9)	
H23	0.0527	0.718	−0.1621	0.025*	
C24	0.0567 (3)	0.4488 (6)	−0.1089 (3)	0.0187 (8)	
H24	0.0164	0.3954	−0.1613	0.022*	
C25	0.0959 (3)	0.3368 (6)	−0.0305 (3)	0.0178 (8)	
H25	0.0819	0.2072	−0.0318	0.021*	
N1	0.1538 (2)	0.4066 (5)	0.0481 (2)	0.0149 (7)	
O1	0.26931 (19)	0.0826 (4)	0.0638 (2)	0.0193 (6)	
O2	0.32142 (19)	0.4095 (4)	0.1875 (2)	0.0192 (6)	
O11	0.3032 (2)	−0.0437 (5)	0.3404 (2)	0.0356 (8)	
O12	0.1301 (2)	0.4540 (4)	0.3090 (2)	0.0274 (7)	
O13	0.0548 (2)	−0.0508 (4)	0.1444 (2)	0.0237 (7)	
F1	0.4843 (2)	0.5667 (5)	0.2568 (2)	0.0587 (10)	
F2	0.3971 (3)	0.7446 (4)	0.1556 (3)	0.0615 (11)	
F3	0.5014 (2)	0.5958 (6)	0.1124 (3)	0.0682 (11)	
Re1	0.213272 (11)	0.21860 (2)	0.171210 (11)	0.01468 (6)	

### Atomic displacement parameters (Å^2^)

**Table:**

	*U*^11^	*U*^22^	*U*^33^	*U*^12^	*U*^13^	*U*^23^
C1	0.023 (2)	0.020 (2)	0.019 (2)	0.0079 (17)	0.0038 (17)	0.0041 (17)
C2	0.017 (2)	0.031 (2)	0.024 (2)	−0.0022 (18)	0.0096 (19)	0.0017 (18)
C3	0.017 (2)	0.022 (2)	0.024 (2)	−0.0005 (17)	0.0029 (18)	0.0071 (17)
C4	0.032 (3)	0.029 (2)	0.029 (2)	0.005 (2)	0.015 (2)	−0.0023 (19)
C5	0.026 (3)	0.031 (2)	0.033 (3)	−0.010 (2)	0.011 (2)	−0.001 (2)
C11	0.022 (2)	0.020 (2)	0.025 (2)	−0.0016 (17)	0.0051 (19)	−0.0013 (17)
C12	0.022 (2)	0.0168 (19)	0.020 (2)	−0.0029 (16)	0.0072 (17)	0.0033 (16)
C13	0.024 (2)	0.0153 (19)	0.015 (2)	0.0023 (16)	0.0068 (17)	−0.0007 (15)
C21	0.021 (2)	0.0140 (18)	0.022 (2)	−0.0033 (16)	0.0064 (18)	−0.0050 (16)
C22	0.025 (2)	0.0113 (17)	0.027 (2)	−0.0011 (16)	0.0108 (19)	−0.0009 (16)
C23	0.026 (2)	0.0166 (19)	0.020 (2)	0.0018 (17)	0.0068 (18)	0.0042 (16)
C24	0.020 (2)	0.021 (2)	0.015 (2)	0.0006 (16)	0.0035 (17)	−0.0022 (15)
C25	0.022 (2)	0.0154 (17)	0.017 (2)	−0.0015 (16)	0.0072 (17)	−0.0024 (16)
N1	0.0179 (18)	0.0149 (15)	0.0124 (16)	−0.0004 (13)	0.0049 (14)	−0.0005 (12)
O1	0.0229 (16)	0.0155 (13)	0.0214 (15)	0.0009 (12)	0.0093 (13)	−0.0006 (11)
O2	0.0173 (15)	0.0203 (14)	0.0205 (15)	−0.0051 (11)	0.0056 (12)	−0.0016 (12)
O11	0.039 (2)	0.0347 (19)	0.0300 (19)	0.0023 (15)	0.0004 (16)	0.0119 (15)
O12	0.036 (2)	0.0215 (15)	0.0304 (18)	0.0001 (13)	0.0198 (15)	−0.0045 (13)
O13	0.0219 (17)	0.0196 (15)	0.0309 (17)	−0.0074 (13)	0.0089 (14)	−0.0033 (13)
F1	0.059 (2)	0.057 (2)	0.050 (2)	−0.0246 (18)	−0.0092 (18)	−0.0007 (17)
F2	0.050 (2)	0.0276 (16)	0.104 (3)	−0.0086 (15)	0.011 (2)	0.0036 (18)
F3	0.060 (2)	0.070 (2)	0.088 (3)	−0.034 (2)	0.045 (2)	−0.019 (2)
Re1	0.01714 (10)	0.01195 (8)	0.01581 (9)	−0.00153 (6)	0.00557 (6)	−0.00083 (6)

### Geometric parameters (Å, °)

**Table:**

C1—O1	1.263 (5)	C13—O13	1.150 (5)
C1—C2	1.409 (6)	C13—Re1	1.906 (4)
C1—C4	1.513 (6)	C21—N1	1.360 (5)
C2—C3	1.369 (6)	C21—C22	1.370 (6)
C2—H2	0.93	C21—H21	0.93
C3—O2	1.269 (5)	C22—C23	1.392 (6)
C3—C5	1.514 (6)	C22—H22	0.93
C4—H4A	0.96	C23—C24	1.377 (6)
C4—H4B	0.96	C23—H23	0.93
C4—H4C	0.96	C24—C25	1.376 (6)
C5—F1	1.298 (6)	C24—H24	0.93
C5—F3	1.317 (5)	C25—N1	1.347 (5)
C5—F2	1.353 (6)	C25—H25	0.93
C11—O11	1.144 (5)	N1—Re1	2.202 (3)
C11—Re1	1.932 (5)	O1—Re1	2.135 (3)
C12—O12	1.143 (5)	O2—Re1	2.117 (3)
C12—Re1	1.906 (4)		
			
O1—C1—C2	125.0 (4)	C24—C23—C22	118.2 (4)
O1—C1—C4	116.3 (4)	C24—C23—H23	120.9
C2—C1—C4	118.7 (4)	C22—C23—H23	120.9
C3—C2—C1	124.6 (4)	C25—C24—C23	119.6 (4)
C3—C2—H2	117.7	C25—C24—H24	120.2
C1—C2—H2	117.7	C23—C24—H24	120.2
O2—C3—C2	129.2 (4)	N1—C25—C24	122.9 (4)
O2—C3—C5	111.3 (4)	N1—C25—H25	118.6
C2—C3—C5	119.5 (4)	C24—C25—H25	118.6
C1—C4—H4A	109.5	C25—N1—C21	117.3 (3)
C1—C4—H4B	109.5	C25—N1—Re1	120.8 (3)
H4A—C4—H4B	109.5	C21—N1—Re1	121.9 (3)
C1—C4—H4C	109.5	C1—O1—Re1	129.3 (3)
H4A—C4—H4C	109.5	C3—O2—Re1	126.7 (3)
H4B—C4—H4C	109.5	C12—Re1—C13	87.55 (17)
F1—C5—F3	108.3 (4)	C12—Re1—C11	90.32 (18)
F1—C5—F2	106.8 (4)	C13—Re1—C11	87.88 (18)
F3—C5—F2	105.9 (4)	C12—Re1—O2	92.70 (14)
F1—C5—C3	111.4 (4)	C13—Re1—O2	178.20 (14)
F3—C5—C3	114.4 (4)	C11—Re1—O2	93.89 (15)
F2—C5—C3	109.7 (4)	C12—Re1—O1	174.14 (14)
O11—C11—Re1	177.7 (4)	C13—Re1—O1	94.59 (14)
O12—C12—Re1	177.4 (3)	C11—Re1—O1	95.20 (15)
O13—C13—Re1	178.3 (4)	O2—Re1—O1	84.99 (11)
N1—C21—C22	122.5 (4)	C12—Re1—N1	91.68 (15)
N1—C21—H21	118.8	C13—Re1—N1	94.55 (15)
C22—C21—H21	118.8	C11—Re1—N1	176.91 (15)
C21—C22—C23	119.6 (4)	O2—Re1—N1	83.67 (12)
C21—C22—H22	120.2	O1—Re1—N1	82.72 (11)
C23—C22—H22	120.2		

### Hydrogen-bond geometry (Å, °)

**Table:**

*D*—H···*A*	*D*—H	H···*A*	*D*···*A*	*D*—H···*A*
C2—H2···F3^i^	0.93	2.55	3.407 (6)	153.
C22—H22···O1^ii^	0.93	2.58	3.360 (5)	142

Symmetry codes: (i) −*x*+1, −*y*+1, −*z*; (ii) *x*, *y*+1, *z*.
